# The Metastable State of Fermi–Pasta–Ulam–Tsingou Models

**DOI:** 10.3390/e25020300

**Published:** 2023-02-06

**Authors:** Kevin A. Reiss, David K. Campbell

**Affiliations:** Department of Physics, Boston University, Boston, MA 02215, USA

**Keywords:** metastability, classical statistical mechanics, advanced numerical methods, semiclassical methods and results

## Abstract

Classical statistical mechanics has long relied on assumptions such as the equipartition theorem to understand the behavior of the complicated systems of many particles. The successes of this approach are well known, but there are also many well-known issues with classical theories. For some of these, the introduction of quantum mechanics is necessary, e.g., the ultraviolet catastrophe. However, more recently, the validity of assumptions such as the equipartition of energy in classical systems was called into question. For instance, a detailed analysis of a simplified model for blackbody radiation was apparently able to deduce the Stefan–Boltzmann law using purely classical statistical mechanics. This novel approach involved a careful analysis of a “metastable” state which greatly delays the approach to equilibrium. In this paper, we perform a broad analysis of such a metastable state in the classical Fermi–Pasta–Ulam–Tsingou (FPUT) models. We treat both the α-FPUT and β-FPUT models, exploring both quantitative and qualitative behavior. After introducing the models, we validate our methodology by reproducing the well-known FPUT recurrences in both models and confirming earlier results on how the strength of the recurrences depends on a single system parameter. We establish that the metastable state in the FPUT models can be defined by using a single degree-of-freedom measure—the spectral entropy (η)—and show that this measure has the power to quantify the distance from equipartition. For the α-FPUT model, a comparison to the integrable Toda lattice allows us to define rather clearly the lifetime of the metastable state for the standard initial conditions. We next devise a method to measure the lifetime of the metastable state $t_{m}$ in the α-FPUT model that reduces the sensitivity to the exact initial conditions. Our procedure involves averaging over random initial phases in the plane of initial conditions, the $P_{1}$-$Q_{1}$ plane. Applying this procedure gives us a power-law scaling for $t_{m}$, with the important result that the power laws for different system sizes collapse down to the same exponent as $E\alpha^{2}\to 0$. We examine the energy spectrum E(k) over time in the α-FPUT model and again compare the results to those of the Toda model. This analysis tentatively supports a method for an irreversible energy dissipation process suggested by Onorato et al.: four-wave and six-wave resonances as described by the “wave turbulence” theory. We next apply a similar approach to the β-FPUT model. Here, we explore in particular the different behavior for the two different signs of β. Finally, we describe a procedure for calculating $t_{m}$ in the β-FPUT model, a very different task than for the α-FPUT model, because the β-FPUT model is not a truncation of an integrable nonlinear model.

## 1. Introduction

Statistical mechanics, broadly speaking, aims to draw conclusions about the behavior of systems with large numbers of particles without needing to solve the even larger number of equations that the system obeys. This approach was successful in explaining everything from the temperature of a gas to the density of a neutron star, with many stunning discoveries in between [1]. One of the central tenets of this subject is the equipartition theorem [2], which assumes that over time, energy will be shared equally around the system. This assumption has led to many successes, e.g., the ideal gas law, but also some failures, e.g., the ultraviolet catastrophe from the failure of the Rayleigh–Jeans law to describe blackbody radiation [3]. Until recently, it was believed that the resolution of the ultraviolet catastrophe required the quantization of the energy of light into photons. However, more recently, an entirely classical resolution was proposed [4]. By avoiding the assumption of the equipartition theorem, Wang et al. were able to find the Stefan–Boltzmann law through purely classical mechanics, consistent with the results of quantum mechanics. The key was the statistics of a quasi-stationary state in the model, which has the effect of stalling the approach to equilibrium. While the impact of these new results on statistical mechanics and the approach to equilibrium remains to be seen, the suggestion that “metastable” states may play a critical role in the interactions of many-body classical systems is very intriguing and is something that we will study in detail in this paper.

As the background and motivation for our study, we recall that, in the early 1950s, Enrico Fermi, John Pasta, Stanislaw Ulam and Mary Tsingou (FPUT) made the first detailed computational study of the validity of the equipartition theorem. For the parameters used in their studies, instead of equipartition, they observed a similar quasi-stationary state consisting of “recurrences” to the initial state [5]. Their assumption was that adding even a small nonlinear term to the linear couplings between harmonic oscillators would be enough to allow the system to thermalize, i.e., reach a state of equipartition. However, they found that for small enough energies, the system remained localized in the mode space for all the times that were computationally possible to explore with their computer. Further, they found that there were remarkable and entirely unexpected (near) recurrences to the initial state. This discovery opened the door for many important advances in the field of nonlinear dynamical systems: the discovery of solitons [6], *q*-breathers [7] and many more. Some of the most significant implications of their results were summarized on its 50-year anniversary [8]. The dedicated reader is referred to these major reviews of the FPUT problem: [9,10,11,12].

Our interest here is to explore computationally what is referred to as the “metastable state” [13] in the FPUT models. This is a quasi-stationary state which stalls the approach to equipartition. In particular, we are interested in the lifetime of the metastable state, because the system is not able to approach equilibrium until the metastable state has ended. Understanding the lifetime of this state, especially any scaling laws that it exhibits, will likely provide a basis for analyzing other systems with quasi-stationary states. Hence, we will develop and explore some techniques which can standardize the study of metastable states in non-integrable systems. It is our belief that the continued exploration of these states in physical systems has the potential to unlock more equivalencies between quantum mechanics and classical statistical mechanics, as was the case with blackbody radiation [4].

The structure of the remainder of this paper is as follows. First, in Section 2, we introduce the systems we will explore. Then, in Section 3, we lay out the recurrence phenomenon and give an intuitive picture of the metastable state. In Section 4, we explore the metastable state in the α-FPUT model, the primary computational focus of our article. In the next section, we explore the strength of the recurrences (Section 4.1), the lifetime of the metastable state (Section 4.2) and the energy spectrum (Section 4.3). We conclude with a qualitative exploration of the metastable state in the β-FPUT model, in Section 5. We examine a comparison between the two signs of β (Section 5.1), and in Section 5.2, we discuss the possibility of measuring the lifetime of the metastable state in the β model. Section 6 presents a summary of our conclusions.

## 2. Methods

### 2.1. Models

The general Hamiltonian for the systems we will consider is that of a chain of oscillators constrained to move in one dimension with nearest neighbor interactions given by a potential V(r), i.e.,

(1) $$H\left(q,p\right)=\sum_{n=1}^{N}\frac{p_{n}^{2}}{2}+\sum_{n=0}^{N}V\left(q_{n+1}-q_{n}\right).$$

We will consider both the α-FPUT model, with a cubic potential,

(2) $$V_{\alpha }\left(r\right)=\frac{r^{2}}{2}+\frac{\alpha }{3}r^{3},$$

and the β-FPUT model, with a quartic potential,

(3) $$V_{\beta }\left(r\right)=\frac{r^{2}}{2}+\frac{\beta }{4}r^{4},$$

with fixed boundary conditions $q_{0}=q_{N+1}=0$ and $p_{0}=p_{N+1}=0$ such that there are *N* distinguishable oscillators. The β-FPUT model can be considered as a perturbation of a linear chain of oscillators (with perturbation strength β), while the α-FPUT model behaves as a truncation of the Toda lattice, which has potential energy:

(4) $$V_{\mathrm{Toda}}\left(r\right)=V_{0}\left(e^{\lambda r}-1-\lambda r\right),$$

and was shown to be completely integrable [14].

We define the normal modes through the canonical transformation:

(5) $$\begin{array}{c} \left[\begin{array}{c} q_{n}\\ p_{n} \end{array}\right]=\sqrt{\frac{2}{N+1}}\sum_{k=1}^{N}\left[\begin{array}{c} Q_{k}\\ P_{k} \end{array}\right]\sin\left(\frac{nk\pi }{N+1}\right). \end{array}$$

These normal modes have frequencies:

(6) $$\omega_{k}=2\sin\left(\frac{k\pi }{2(N+1)}\right).$$

This normal mode transformation diagonalizes the harmonic lattice (i.e., α=β=0 only) but leaves off-diagonal terms in the Hamiltonians for the anharmonic models (α,β≠0). These terms lead to the transfer of energy among the modes.

After this normal mode transformation, the Hamiltonian for the α-FPUT model is

(7) $$H_{\alpha }\left(Q,P\right)=\sum_{k=1}^{N}\frac{P_{k}^{2}+\omega_{k}^{2}Q_{k}^{2}}{2}+\frac{\alpha }{3}\sum_{k,j,l=1}^{N}A_{k,j,l}Q_{k}Q_{j}Q_{l},$$

while for the β-FPUT:

(8) $$H_{\beta }\left(Q,P\right)=\sum_{k=1}^{N}\frac{P_{k}^{2}+\omega_{k}^{2}Q_{k}^{2}}{2}+\frac{\beta }{4}\sum_{i,j,l=1}^{N}B_{k,i,j,l}Q_{k}Q_{i}Q_{j}Q_{l},$$

where the last (summed) terms in both equations couple the normal modes together, allowing for energy sharing, with coupling constants given by [15,16]:

(9) $$A_{k,j,l}=\frac{\omega_{k}\omega_{j}\omega_{l}}{\sqrt{2(N+1)}}\sum_{\pm }\left[\delta_{k,\pm j\pm l}-\delta_{k\pm j\pm l,2(N+1)}\right],$$

(10) $$B_{k,i,j,l}=\frac{\omega_{k}\omega_{i}\omega_{j}\omega_{l}}{2(N+1)}\sum_{\pm }\left[\delta_{k,\pm j\pm l\pm m}-\delta_{k\pm j\pm l\pm m,\pm 2(N+1)}\right],$$

where $\delta_{i,j}$ is the Kronecker delta function and the sums $\sum_{\pm }$ are overall combination of plus and minus signs in the equation.

The energy $E_{k}$ of the *k*-th mode is

(11) $$E_{k}=\frac{1}{2}\left(P_{k}^{2}+\omega_{k}^{2}Q_{k}^{2}\right).$$

This definition is exact only for the harmonic lattice, but serves as a good approximation for weak nonlinearity, because any contributions to the energy coming from coupled modes have a pre-factor of the nonlinear strength (α or β).

Whenever a quantity is time-averaged, we place a line over its symbol (e.g., $\bar{E}$). This represents a time average from time t=0 to t=T, i.e.,

(12) $$\bar{E}\left(T\right)=\frac{1}{T}\int_{0}^{T}E\left(t\right)\;dt.$$

### 2.2. Numerical Methods

For integrations involving the α-FPUT model and β-FPUT model with β<0, which were observed to be reasonably stable numerically [17], we use the $SABA_{2}C$ symplectic integration scheme described in appendix 1 of [18]. This scheme has error $O({\left[dt\right]}^{4})$. For the integration of the Toda lattice, we use the $SABA_{2}$ scheme, i.e., the same scheme but without the corrector Hamiltonian term, giving error $O({\left[dt\right]}^{2})$, which was determined to provide sufficient accuracy for the range of parameters considered. For β>0, the β-FPUT model is known to exhibit exponential numerical instabilities related to instabilities of the soliton solutions to the modified Korteweg–de Vries (mKdV) equation [19], because the mKdV equation arises from the continuum limit of the β-FPUT model. To reduce the need for extremely small time-step sizes, we implement the symplectic integrator $SABA_{2}Y8\_D$ described in [20] and in Table 2 of [21], which has error $O({\left[dt\right]}^{8})$. In general, we use a time step dt=0.1 unless a failure of time reversal requires us to decrease dt to improve the accuracy.

### 2.3. Spectral Entropy

We will use spectral entropy to quantify the FPUT system’s “distance” from equipartition at a given time. The spectral entropy is similar to Shannon information entropy [22] and is defined as

(13) $$\begin{array}{c} S\left(t\right)=-\sum_{k=1}^{N}e_{k}\left(t\right)\ln\left[e_{k}\left(t\right)\right],\\ \mathrm{with}:\;e_{k}\left(t\right)=\frac{E_{k}\left(t\right)}{\sum_{k}E_{k}\left(t\right)}, \end{array}$$

where $e_{k}\left(t\right)$ is the proportion of linear energy in mode *k* at time *t*. Spectral entropy ranges from 0, when all the energy is in one mode, to $S_{max}$, when an equal amount of energy is present in all modes. For the α-FPUT and Toda lattices, equal energy sharing corresponds to $e_{k}=1/N$∀*k*; therefore, $S_{max}=\ln\left(N\right)$. However, the β-FPUT lattice remains symmetric about its center for initially symmetric excitations, and therefore energy cannot spread from an even-numbered mode number to an odd-numbered mode, or vice versa. Because our initial conditions will include only an odd mode, energy can only be shared among odd modes, so $S_{max}=\ln\left\lceil \frac{N}{2}\right\rceil$, where ⌈⌉ is the ceiling function, which rounds a number up to the next highest integer. Because this definition of spectral entropy has a different maximum value for different lattice sizes *N*, we can rescale it by defining the rescaled spectral entropy (henceforth entropy for short):

(14) $$\eta \left(t\right)=\frac{S\left(t\right)-S_{max}}{S\left(0\right)-S_{max}}.$$

This is a convenient definition because η ranges from 1 at t=0 to 0 when energy is shared equally among all modes (equipartition), regardless of system size *N*.

## 3. Phenomena

### 3.1. FPUT Recurrences

**Figure 1:**
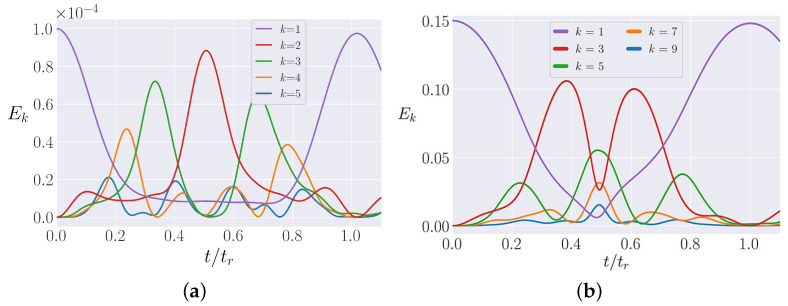
The energy in each normal mode in the α-FPUT and β-FPUT models as a function of time. At $t=t_{r}$, the first FPUT recurrence is observed, with nearly all energy returning to its initial condition, the first normal mode. The lowest 5 allowed modes in each model are plotted. (**a**) α-FPUT model with initial $E_{1}=10^{-4}$ and N=127 (with α=1). (**b**) β-FPUT model with initial $E_{1}=0.15$ and N=127 (with β=1).

One of the surprising features of the models first explored by FPUT [5] was the presence of what have come to be known as “FPUT recurrences”. Indeed, Fermi himself expressed the (understated) opinion that this behavior really constituted a “little discovery in providing limitations that the prevalent beliefs in the universality of “mixing” and “thermalization” in nonlinear systems may not always be justified” [5]. The FPUT recurrences were discovered as follows: when all of the energy was initialized in the first normal mode, this energy was first observed to diffuse to higher order modes, but then the energy began to return to the first normal mode, eventually nearly fully returning at what is called the “recurrence time” ($t_{r}$). This phenomenon is shown in Figure 1, which shows the energy in the lowest 5 allowed modes in the α-FPUT and β-FPUT models as a function of time. As noted above, the β-FPUT lattice preserves the symmetry about its center so with the initial energy only in mode 1, only odd modes are allowed. At $t=t_{r}$, the systems have nearly reproduced their initial conditions. $t_{r}$ is calculated for the α-FPUT model following [23] and for the β-FPUT model following [24]. The timescale for this recurrent behavior is many orders of magnitude shorter than the Poincaré recurrence time [25], and the recurrences continue quasi-periodically for a long time; indeed, the initial conditions considered by FPUT have yet to be driven to equipartition in any computer simulation. However, for larger initial energy, the FPUT recurrences eventually breakdown, and the system is able to thermalize. Clearly, when most of the energy is quasi-periodically returning near the initial condition, which is extremely localized, the system remains localized while these recurrences continue to occur.

FPUT recurrences were used to study ultra-cold Bose gases [26], the nonlinear Schrodinger equation [27] and electron–phonon interactions [28], to mention a few applications. Their study was also extended to higher-order recurrences, such as super-recurrences [18,29]. Their existence was explained in various ways, most notably (1) by using *q*-breathers [7,30,31] or (2) by the presence of solitons in the KdV (mKdV) equation, which is the continuum limit of the α-FPUT (β-FPUT) model [6,24,32]. The importance of FPUT recurrences is difficult to overstate, but in this paper, we focus primarily on their role in delaying the approach to equipartition.

### 3.2. Metastable State

The recurrence phenomenon has the effect of stalling the approach to equilibrium by keeping the system’s energy localized near its initial condition. This phenomenon was interpreted, as early as 1982 [33], as the system having two distinct “regions” in time: in the first region, the system relaxes into an intermediate quasi-stationary state, which persists for some time, before it again relaxes, this time into its true equilibrium state defined by equipartition, such that $\bar{\eta }=\langle \eta \rangle$. The intermediate or “metastable” state was more recently studied extensively by Giancarlo Benettin [13,34,35,36]. His work frames the phenomenon as a cross-over between predominantly integrable dynamics to the true non-integrable dynamics of the FPUT models.

**Figure 2:**
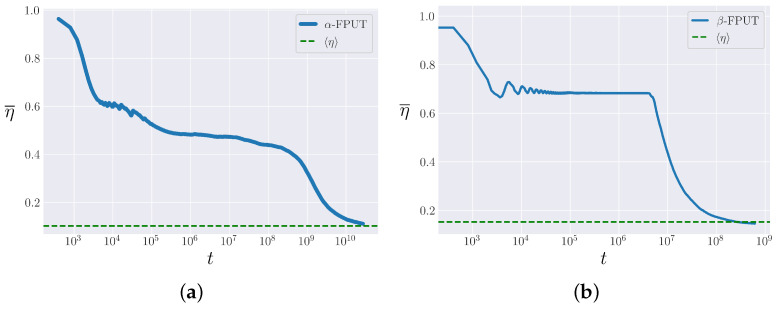
The time-averaged entropy as a function of time in the α-FPUT and β-FPUT models. The ensemble average $\langle \eta \rangle$ (from Equation (15)) is plotted and the agreement $\bar{\eta }=\langle \eta \rangle$ appears to be stalled by a metastable state. (**a**) α-FPUT model with $E\alpha^{2}=0.02$ and N=63. (**b**) β-FPUT model with Eβ=0.57 and N=31.

In terms of the spectral entropy η, the system is considered to be in equilibrium when $\bar{\eta }=\langle \eta \rangle$, where we calculate $\langle \eta \rangle$ following Danieli [37]:

(15) $$\langle \eta \rangle =\frac{1-\gamma }{S_{max}-S\left(0\right)},$$

where γ≃0.577 is the Euler–Mascheroni constant. We are interested in the time that the metastable state persists, before its ultimate destruction, and the system’s approach to equilibrium. We call this the lifetime $t_{m}$ of the metastable state. In Figure 2, we illustrate the metastable state in the α-FPUT and β-FPUT models. We can see that their behaviors are qualitatively quite different. While the α-FPUT model appears to be decreasing gradually in $\bar{\eta }$, the β-FPUT model exhibits a clear flat plateau for a long time before some mechanism causes the metastable state to collapse fairly suddenly. The features of the metastable state in the α-FPUT model, at first glance, make it difficult to define where the metastable state ends and the approach to equilibrium begins, but we will show that we can separate these two regions by comparing the α-FPUT model’s behavior to that of the Toda lattice and considering the crossover time $t_{m}$ to be that time at which the behavior of the two systems begins to differ substantially.

To make this point more explicitly, we note that up to $O(r^{4})$, the α-FPUT potential (Equation (2)) can be thought of as a truncation of the Toda potential (Equation (4)), through a convenient change in the parameters. By setting $V_{0}=\lambda^{-2}$ and λ=2α, and Taylor expanding the Toda potential around r=0, we obtain the following series expansion:

(16) $$V_{\mathrm{Toda}}\left(r\right)=\frac{r^{2}}{2}+\frac{\alpha }{3}r^{3}+\frac{\alpha^{2}}{6}r^{4}+\frac{\alpha^{3}}{15}r^{5}+O\left(r^{6}\right)=V_{\alpha }\left(r\right)+O\left(r^{4}\right).$$

**Figure 3:**
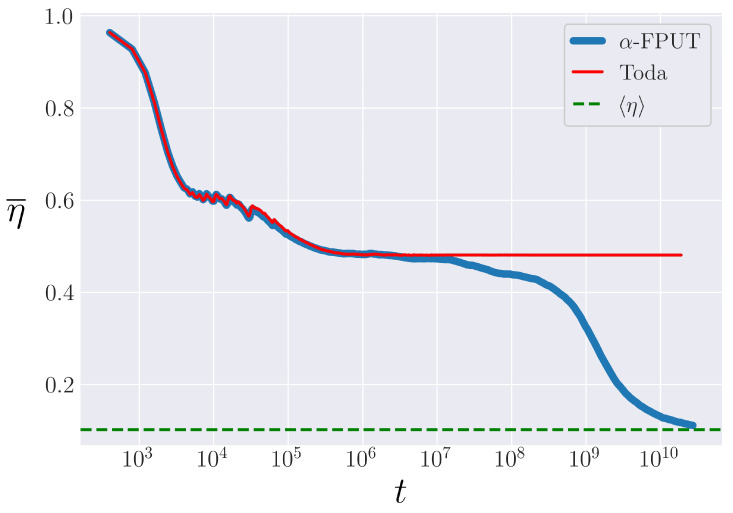
The time-averaged entropy $\bar{\eta }$ as a function of time (note logarithmic time scale) for both the Toda model (red) and α-FPUT model (blue). Both have initial energy $E\alpha^{2}=0.02$ and system size N=63.

Thus, the α-FPUT model’s metastable state can be analyzed by considering its behavior to be similar to the integrable Toda lattice, before it breaks off and exhibits the expected behavior of non-integrable systems [34]. Figure 3 demonstrates the similarity of the evolution of $\bar{\eta }$ between the Toda model and the α-FPUT model up to a certain point in time, after which the α-FPUT model falls to the expected equilibrium value of $\bar{\eta }$: the ensemble average $\langle \eta \rangle$. This comparison to the Toda lattice will allow us to define rather precisely $t_{m}$ in the α-FPUT model.

## 4. α-FPUT Model

### 4.1. Strength of FPUT Recurrences

It was shown [23] that the time to the first FPUT recurrence ($t_{r}$) in the α-FPUT model scales as a function of an essential system parameter *R*, defined as:

(17) $$R={\left(N+1\right)}^{3/2}\sqrt{E\alpha^{2}}.$$

Specifically, as was shown in [23], by rescaling the FPUT recurrence time by ${\left(N+1\right)}^{3}$, then for R≥10:

(18) $$\frac{t_{r}}{{\left(N+1\right)}^{3}}=R^{-1/2}.$$

**Figure 4:**
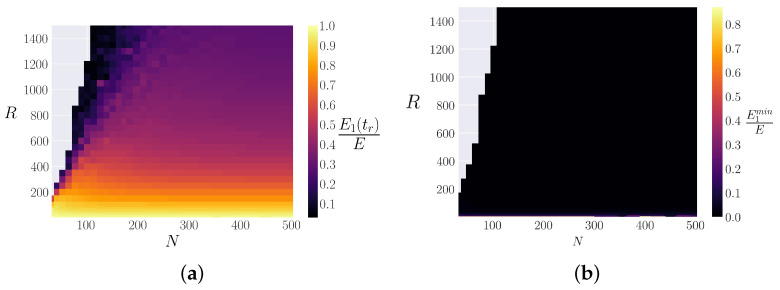
Heatmaps as a function of *R* (Equation (17)) and system size *N* in the α-FPUT model. Note that the gray region corresponds to initial conditions which blow up (potential V(r)→−∞) before $1.5t_{r}$. (**a**) The “strength” of FPUT recurrences, represented by $E_{1}\left(t_{r}\right)/E$, the fraction of energy returning to the initial condition at the first recurrence. (**b**) The quantity $E_{1}^{min}/E$, which represents the proportion of energy that leaves the 1st normal mode before the first recurrence.

We use this expected value of the first FPUT recurrence time and look in the region $0.5t_{r}<t<1.5t_{r}$ for the maximum value of the energy in the first normal mode, and name that $E_{1}\left(t_{r}\right)$. We can then calculate the ratio of this energy to the initial energy and use this as a measure of the relative “strength” of the FPUT recurrence for a given value of *R* and *N*. The results are plotted in Figure 4a and demonstrate that the FPUT recurrence strength drops off as *R* increases, nearly independent of system size *N*.

Although this discussion is similar in motivation to that describing the behavior of the β-FPUT model (see Section 7 of [24]), the implications are quite different: for the β-FPUT model, FPUT recurrences lose strength as a function of the parameter Eβ independent of *N*—**NOT** the essential system parameter

(19) S=Eβ(N+1),

which the FPUT recurrence time scales with. For the α-FPUT model, the strength of the FPUT recurrences scales with *R*, independent of *N*, instead of the corresponding energy parameter $E\alpha^{2}$. It is also worth noting that while in [24] one had to define a parameter called “shareable energy” to compare the quality of the FPUT recurrences between the cases β>0 and β<0, Figure 4b shows that this is not necessary for the α-FPUT model. This figure plots the quantity:

(20) $$\frac{E_{1}^{min}}{E}\equiv \min_{0<t<t_{r}}\frac{E_{1}\left(t\right)}{E},$$

which quantifies how much energy leaves the first normal mode (the initial condition), before most of it comes back at the recurrence time. Figure 4 demonstrates that for the α-FPUT model, nearly all of the energy consistently leaves the first normal mode before coming back for a recurrence. This appears to be true for all *R* and *N*, except in the harmonic limit ($E\alpha^{2}\to 0$). However, this sharing of energy is not the case for the β-FPUT model with β<0, where roughly 70% of the energy remains in the first normal mode before a recurrence [24].

**Figure 5:**
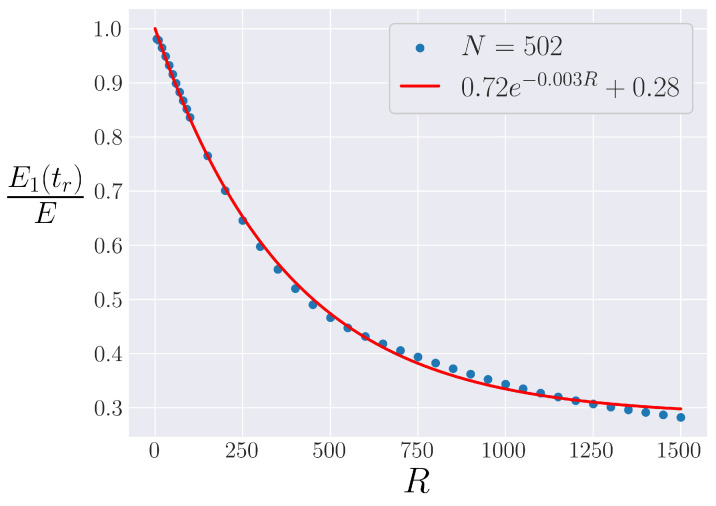
The “strength” of recurrences in the α-FPUT model, $E_{1}\left(t_{r}\right)/E$ as a function of system parameter *R* at fixed N=502. An exponential fit is added.

Because Figure 4a seems to show that the strength of recurrences falls off as a function of *R*, independent of *N* outside of regimes where blow-up is likely, it helps to look at systems with the same *N* and plot the recurrence strength $E_{1}\left(t_{r}\right)/E$ as a function of system parameter *R*. This is performed in Figure 5, and a nearly exponential decay is found. This result holds for all sufficiently large system sizes, and the results are presented for N=502 to avoid blow-up and other small *N* behavior [38] (following [39], we only work with systems such that N+1 is a power of 2 or prime). This exponential decay is again in contrast to the β-FPUT model, where recurrence strength appears to be roughly consistent until a cut-off energy Eβ where the recurrence strength falls precipitously [24].

### 4.2. Lifetime of Metastable State

#### 4.2.1. Procedure

**Figure 6:**
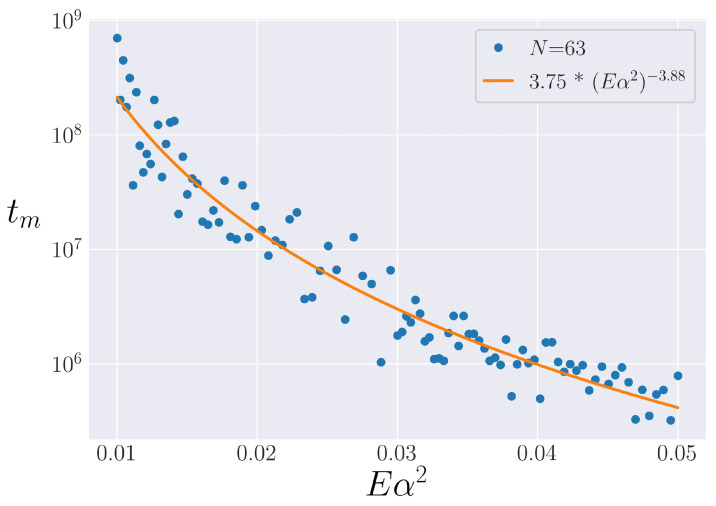
An attempt at defining $t_{m}$ in the α-FPUT model for N=63 by defining an arbitrary tolerance and waiting for a deviation from the Toda model beyond this tolerance. A power-law best fit is added. Note the logarithmic scaling on the $t_{m}$ axis.

We endeavor to find a scaling for the lifetime of the metastable state through a direct comparison to the Toda model, as motivated by Section 3.2. To define $t_{m}$ by comparing the α-FPUT model’s behavior with that of the Toda lattice, the most natural approach is to define some arbitrary tolerance and look for the last time which the α-FPUT model’s entropy is within that tolerance of the entropy of the Toda model. Doing so, however, reveals an interesting feature of the metastable state. The results of following this procedure for N=63 are shown in Figure 6. Even though a clear power-law scaling emerges, the data are quite noisy around this scaling. This noisiness appears to be an inherent feature of the chaotic nature of the α-FPUT model around the metastable state.

Note that because the Toda model is integrable, its dynamics can, in theory, be broken down into actions that remain constant in time and angles that evolve periodically in time. The picture of the metastable state of the α-FPUT model presented by Benettin et al. in [36] is that there are two time scales in the system. In the first one, the actions of the Toda model remain nearly constant even in the α-FPUT model, while the corresponding angles evolve on tori, leading to a behavior very similar to that of the Toda model. Eventually, on a longer time scale, the Toda-like actions in the α-FPUT model start to diffuse throughout the phase space, eventually leading to ergodicity and equipartition. The shorter time scale where the Toda-like actions remain nearly constant defines the metastable state. An important aspect of the transition to diffusing actions is that this diffusion behaves chaotically, with positive definite maximal Lyapunov exponents as described in [36]. This leads to an exponential sensitivity to initial conditions when the diffusion of action variables dominates the dynamics, which explains the noise in Figure 6. To quantify the effect of the initial conditions, we next conduct bin averaging over the initial conditions.

Note that from Equation (11), the energy initially given to the first normal mode can be distributed either in a canonical position or momentum. We define the “phase” θ between our canonical coordinates as:

(21) $$\theta =\tan^{-1}\left(\frac{P_{1}\left(t\right)}{\omega_{1}Q_{1}\left(t\right)}\right).$$

**Figure 7:**
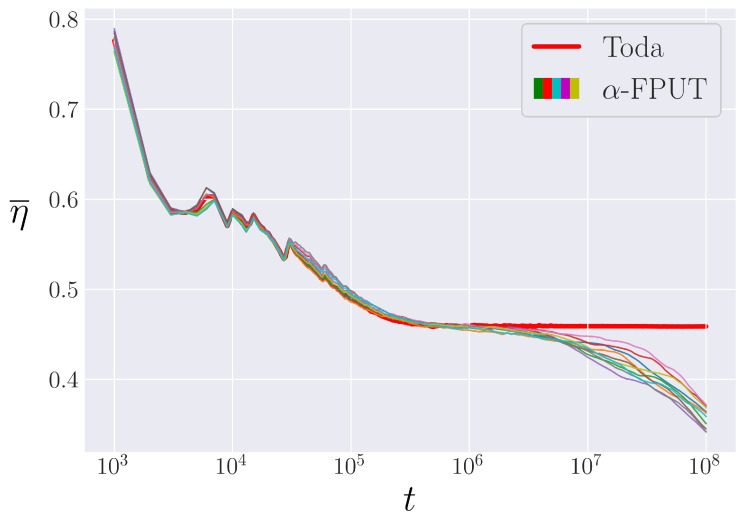
The time-averaged entropy in the Toda model (red curve) compared to 10 bins of α-FPUT trajectories, each made up of the average of 10 random phases. All systems are fixed at energy $E\alpha^{2}=0.028$ and system size N=63.

We can then initialize systems with the same condition $E_{1}\left(0\right)=E$, i.e., the same point in energy space, but slightly separated in the phase space by distributing along the oval of the canonical coordinates defined by rotating θ. In the following, we take 100 random phases for every choice of energy and bin them together to create 10 bins which are each the average of 10 trials with different phases. This bin averaging is meant to calculate an approximation to the ensemble average. An example of the results of this procedure is shown in Figure 7, where each α-FPUT curve represents an average over 10 random phases. Figure 7 demonstrates that each of the α-FPUT trials remains close to the Toda model, up until some time where the entropy starts to decrease below the Toda model entropy (red curve), and then the α-FPUT trajectories start to diverge, not only from the Toda trajectory but also from each other.

We gain two advantages from binning in this manner: (1) we now have a natural length to use as a tolerance cut-off to define the separation between the α-FPUT model and Toda model that is not arbitrary: the standard deviation of each bin; and (2) averaging over 10 different bins again gives us an error bar on our measurement of $t_{m}$ for a given enerzgy. Our procedure is now as follows: Take 10 trials for the α-FPUT model with random phases and average their entropy together. Find the last time that the Toda model’s entropy was within one standard deviation of this bin average. Repeat this for 10 total bins, and average those times together to obtain a measurement of $t_{m}$ with an error bar.

**Figure 8:**
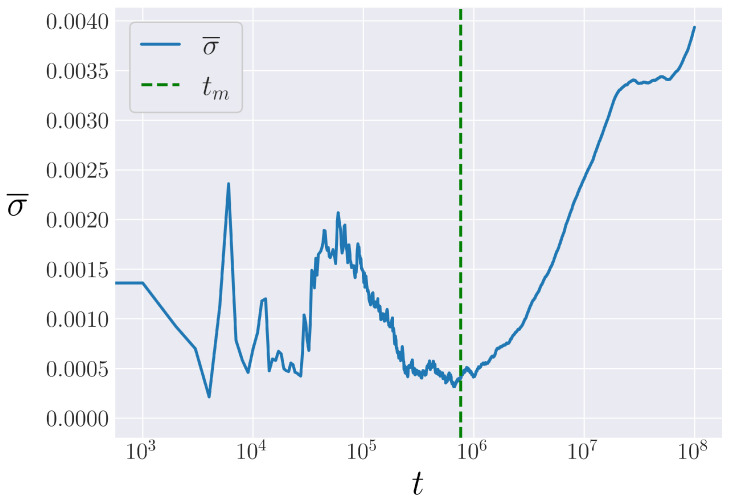
The bin deviation, averaged over bins, again for $E\alpha^{2}=0.028$ and N=63 in the α-FPUT model. The measure value for $t_{m}$ is marked as a vertical, dashed green line.

Performing this operation reveals a surprising result, shown in Figure 8. If we look at the bin standard deviation (σ), averaged over bins ($\bar{\sigma }$), there is a feature similar to a phase transition in the plot. The time at which this occurs happens to line up with the time $t=t_{m}$ as defined in our above procedure. Because our procedure looks at when $\bar{\eta }$ in the α-FPUT model is greater than σ outside of $\bar{\eta }$ in the Toda model, this means $\bar{\eta }$ is falling more quickly than σ is rising in Figure 8, which is significant. This also further validates the point of view that $t_{m}$ represents a transition from mostly integrable dynamics to chaotic, non-integrable dynamics. The growth in $\bar{\sigma }$ for $t>t_{m}$ shows that initially nearby systems are deviating in time, whereas for $t<t_{m}$, $\bar{\sigma }$ is seen to be relatively bounded in time. This also serves to validate our procedure to measure $t_{m}$.

#### 4.2.2. Analysis

**Figure 9:**
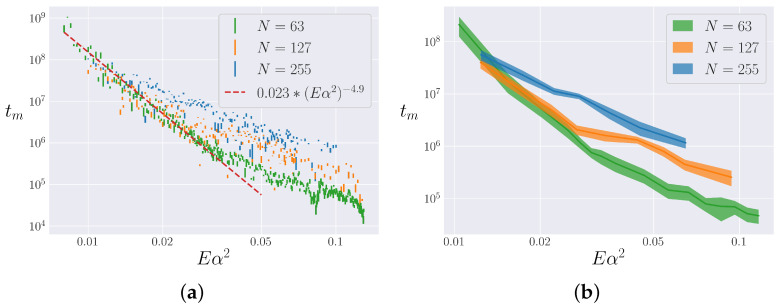
The lifetime of the metastable state as a function of $E\alpha^{2}$ for N=63,127, and 255. Note logarithmic scale on all axes. (**a**) The height of each data point represents its bin error. In the $E\alpha^{2}\to 0$ limit, the data are seen to follow a power law, in dashed red. (**b**) Nearby energies are binned to obtain an upper bound on the noise as a combination of phase and energy error.

We apply the procedure described in the previous section and iterate across a range of energies, for N=63,127 and 255. We determined that system size N=31 was too small and gave erratic results incompatible with the thermodynamic limit. For a discussion of small system size effects in the α-FPUT model, see [38]. We chose system sizes such that N+1 is a power of 2, to avoid resonances discussed in [39]. The results are shown in Figure 9a. The length of each data point is the extent of its bin error. Each system size appears to follow its own trend for high energies. However, for low energies, the data appear to overlap, *regardless of system size.* In this regime, $t_{m}$ is seen to follow a power law, roughly consistent with an exponent of −4.9, as shown by the red dashed line in Figure 9a. A few simulations indicate that this overlap and scaling is consistent for larger system sizes as well. This result is more significant than that presented in Figure 6, as it both considers the ensemble average and appears to hold in the thermodynamic limit. In particular, this result has significant implications for the $E\alpha^{2}\to 0$ limit, which is that originally considered by FPUT.

One interesting aspect of Figure 9a is that the error in the noise (shown by the scattering of the data) seems to be larger than the error due to phase averaging and binning (shown by the height of the data points). In order to account for these two possible sources of chaotic noise, we bin data into groups of 20 consecutive energies to estimate the noise in the energy. Then, we assume that the phase noise ($\sigma_{\theta }$) and energy noise ($\sigma_{E}$) are independent and add them together as

(22) $$\sigma =\sqrt{\sigma_{\theta }^{2}+\sigma_{E}^{2}},$$

to perform the error propagation and obtain an upper bound on the noise. The results are presented in Figure 9b. This gives a better idea of the noise (inherent because the metastable state signals the onset of chaos) in the lifetime of the metastable state.

### 4.3. Spectrum

**Figure 10:**
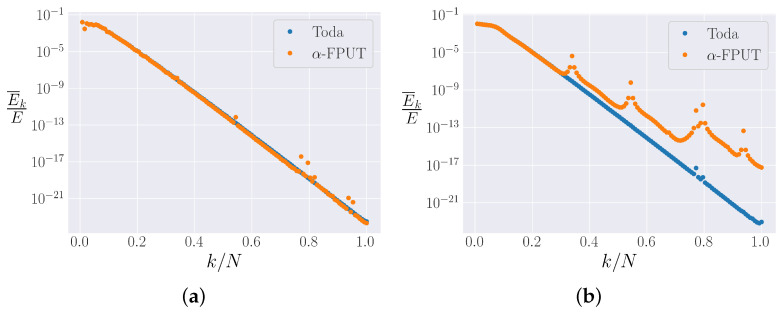
The spectra of the α-FPUT and Toda models for system parameter R=75 and N=127. Spectra are compared at two different times. (**a**) Spectra for $t=10^{5}$. (**b**) Spectra for $t=10^{8}$.

Our use of spectral entropy as the single measure of the destruction of the metastable states gives a qualitative picture, but by plotting the time-averaged energy in each normal mode, at a given time, we have access to many more degrees of freedom than simply looking at the entropy. Therefore, we can obtain a more complete picture. For short times, we expect the spectra of the α-FPUT and Toda models to look essentially identical. This is indeed the case. As time goes on, however, the Toda spectrum flattens out to an exponential tail, which is the shape of the α-FPUT spectrum in the metastable state as well. Nonetheless, some higher modes start to gain energy and spread this energy to the other nearby modes. This process continues until most higher modes are excited and the system approaches equipartition. This behavior is demonstrated in Figure 10. For more discussion on the spectral picture of diffusion in the α-FPUT model compared to the Toda lattice, see [40].

Figure 10a is plotted at $t=10^{5}$ and shows that the spectra of the α-FPUT model and the Toda model largely agree at this time. Figure 10b is plotted at $t=10^{8}$, and we can see that resonances have caused local peaks in the α-FPUT spectrum, which diffuse the energy into the modes around them. This has the effect of lifting the spectrum at each resonance, a process which continues until the system reaches equipartition. In [41], Onorato et al. showed that four-wave resonances in the thermodynamic limit of the α-FPUT model lead to irreversible energy mixing. It was also shown that six-wave interactions are always possible and lead to irreversible energy mixing. Despite the appealing possibility that the observed peaks in the spectra might correspond to those predicted by the wave turbulence method of Onorato et al., we have at present been unable to verify this possibility quantitatively. It is possible that the two largest peaks in the spectra of the α-FPUT model (Figure 10b) are actually made of two resonant modes each, so it is unclear if this is an example of a four-wave or six-wave resonance. We are currently investigating this matter further. Another peculiarity in Figure 10b is the apparent high-k modes in the Toda model which lie well above an exponential tail, even after a long time. The peaks around k/N≃0.8 do not appear to be a numerical artifact, so there could possibly be resonances in the integrable limit (which do *not* lead to irreversible energy mixing).

## 5. β-FPUT Model

### 5.1. Comparison between β>0 and β<0

**Figure 11:**
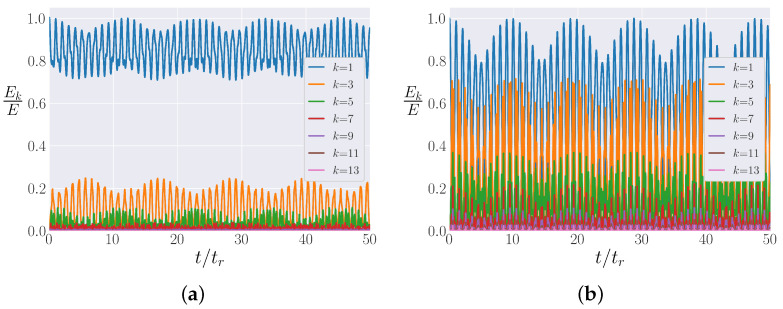
The energy in the lowest 13 modes as a function of time for N=127, and the two choices of the sign of β, in the β-FPUT model when the initial energy is all in the first mode. Recall that in the β model, this means that all even modes have zero energy. Energy in each mode is rescaled by initial energy *E* and time is rescaled by the FPUT recurrence time $t_{r}$. (**a**) Eβ=−0.35 (Note: β<0). (**b**) Eβ=0.15 (Note: β>0).

**Figure 12:**
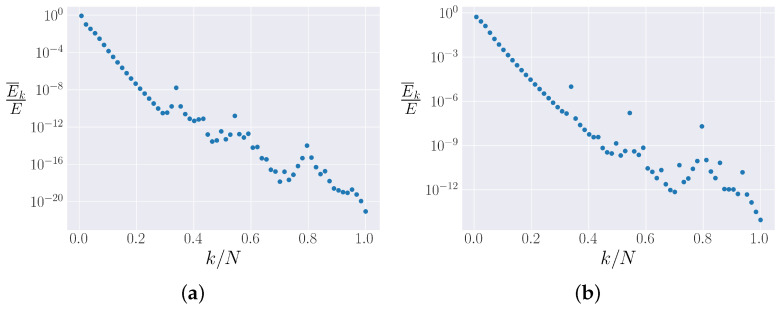
Time-averaged energies in each mode for N=127, as a function of mode number *k*. The average is taken after 50 FPUT recurrences have taken place. (**a**) Eβ=−0.35 (Note: β<0). (**b**) Eβ=0.15 (Note: β>0).

A surprising difference between the FPUT recurrences in the β-FPUT model for the different signs of β was noted in Section 7 of [24]. The difference is qualitatively demonstrated in Figure 11a (β<0) and Figure 11b (β>0), which show the proportion of the total energy in each of the first 13 modes against time. The results are plotted for the first 50 FPUT recurrences, with the FPUT recurrence time $t_{r}$ calculated using the results from [24]. For a system with β>0, the energy almost entirely leaves the first normal mode before coming back at an FPUT recurrence (as demonstrated by Figure 1, which is essentially a zoom into Figure 11b). When β<0, nearly 70% of the energy always remains in the first normal mode during the metastable state. Figure 12b shows that the first normal mode is not isolated when β>0 for the relatively small Eβ=0.15, driving the magnitude of Eβ higher for β<0, as shown in Figure 12a for Eβ=−0.35, which leaves the first normal mode still largely isolated. Figure 6 of [24] shows that this behavior is only a function of the sign of Eβ, not its magnitude.

When the distribution of energy among all the normal modes (energy spectrum) in the metastable state is considered, however, the two systems are relatively similar. Figure 12a (β<0) and Figure 12b (β>0) plot the spectra of the two β-FPUT systems, i.e., the time-averaged energy in each mode. The time averages are computed after 50 FPUT recurrences have occurred. Both spectra follow an exponential decay, with a few peaks in the spectra raising further questions. In [41], Onorato et al. showed that six-wave resonances lead to irreversible energy mixing; these peaks might correspond to those resonances. This possibility is under further investigation.

Figure 12 shows that the differences noted in Figure 11 are only evident between the 1st and 3rd normal modes, with all the other modes following a qualitatively similar distribution. It is possible that for β<0, the k=1 mode engages in the energy-diffusing resonance while k=1 is not a resonant mode for β>0. This would explain the lack of energy mixing for β<0 and the local peak at k=1 in the spectrum (Figure 12a).

### 5.2. Lifetime of Metastable State

As depicted in Figure 2, the metastable state in the β-FPUT model ends much more abruptly than that in the α-FPUT model. However, the α-FPUT model can be considered a truncation of the integrable Toda lattice, so that the point at which the α-FPUT spectral entropy begins to deviate substantially from that of the Toda model is well defined for any set of parameters and can be considered as the end of the metastable state. We showed how to make this even more precise by taking bin averages that effectively approximate an ensemble average. We find that this is very well defined for all the ranges of parameters we have studied in the α-FPUT model. In contrast, the β-FPUT model cannot be viewed as the truncation of any nonlinear integrable model but rather as a perturbation of the linear lattice. This observation coupled with the fact that for β>0, the β-FPUT model exhibits well-known exponential numerical instabilities related to the soliton solutions of the modified Korteweg–de Vries (mKdV) equation [19], (see Section 2.2), results in significant convergence issues when we try to adapt our “bin averaging” technique for the β-FPUT model. In particular, it requires very accurate numerics to ensure that we are correctly following the true dynamics of a given trajectory over time in the β-FPUT model, because there are no non-trivial integrable models with which to compare. If we look again at Figure 2 in Section 3.2, these comments seem counter-intuitive, because the α-FPUT model seems to “slide” down from the metastable state to true equilibrium, whereas the β-FPUT model shows a sudden drop-off to the equilibrium value of the spectral entropy. However, in the β-FPUT model, using the binning procedure to establish the true lifetime of the metastable stage requires an enormous number of runs of very high accuracy to ensure that we are not observing a numerical artifact, i.e., an inaccurate calculation of the true trajectory. To determine a fit for $t_{m}$ against $E\alpha^{2}$, shown in Figure 9 for the α-FPUT model, roughly 20,000 simulations were needed for each system size. With the aforementioned computational difficulties in the β-FPUT model, this computation is exceedingly expensive.

**Figure 13:**
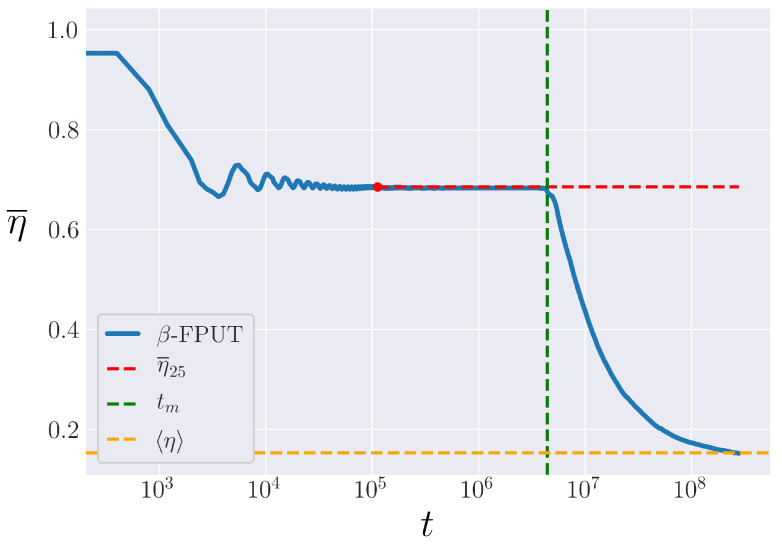
The entropy of the β-FPUT system for N=31, Eβ=0.57. The calculated value of $\bar{\eta }$ in the metastable state, ${\bar{\eta }}_{25}$, is shown starting at the time of the 25th recurrence as a red dashed line, and the calculated metastable lifetime, $t_{m}$, shown as a vertical green line. The ensemble average, $\langle \eta \rangle$, is shown in orange.

We will endeavor nonetheless to describe a possible procedure for calculating how $t_{m}$ in the β-FPUT model scales as a function of Eβ, which can be undertaken in future works. As in the α-FPUT model, we run 100 trials with random initial phases and bin them into 10 bins with 10 trials each. We then define ${\bar{\eta }}_{25}$, the time-averaged entropy after 25 FPUT recurrence times, averaged across the 10 trials in a bin. The recurrence time is calculated following [24]. The number 25 was chosen because this allows the system to relax into its metastable state. After ${\bar{\eta }}_{25}$, then $\bar{\eta }$ tends to be nearly constant in time up until a critical time where it starts relaxing to equilibrium. We can find this critical time, $t_{m}$, by calculating the deviation of $\bar{\eta }$ from ${\bar{\eta }}_{25}$ by more than the bin standard deviation of the 10 trials. This procedure is visualized in Figure 13, where ${\bar{\eta }}_{25}$ is plotted in red, and the computed $t_{m}$ is seen to line up with the end of the plateau in $\bar{\eta }$. Finally, averaging this result over the 10 bins gives a measurement of $t_{m}$ with associated error bars.

## 6. Conclusions

In this article, we have investigated the metastable state in the α-FPUT and β-FPUT models, both qualitatively and quantitatively. We began with a visualization of the metastable state using spectral entropy (η). This single-degree-of-freedom measure has the power to quantify the distance from the equipartition. This approach allowed us to follow G. Benettin [34] in viewing the α-FPUT model as a truncation of the integrable Toda model.

We next studied the strength of the recurrences in the α-FPUT model, following the results from S. Pace [24] on the β-FPUT model. This yielded the surprising result that the recurrence strength is a function only of the essential system parameter $R={\left(N+1\right)}^{3/2}\sqrt{E\alpha^{2}}$ in the α-FPUT model, whereas the strength of the recurrences in the β-FPUT model scale with the energy Eβ and not the essential system parameter S=Eβ(N+1). The strength of the recurrences was shown to decay exponentially with *R*, independent of the system size *N*.

We devised a method to measure the lifetime of the metastable state $t_{m}$ in the α-FPUT model. Our procedure involved averaging over the random initial phases in the $P_{1}$-$Q_{1}$ plane (at fixed energy $E_{1}=\frac{1}{2}\left(P_{1}^{2}+\omega_{k}^{2}Q_{1}^{2}\right)$). This bin average provided a relevant length distance, the standard deviation, from which we could determine when the α-FPUT model trajectories break off from the entropy of the Toda model. Applying this procedure yielded Figure 9a, which shows $t_{m}$ as a function of $E\alpha^{2}$ for different *N*. Surprisingly, as $E\alpha^{2}\to 0$, the data for the different *N* collapse onto the same power law with the exponent −4.9.

We also explored the spectrum of the α-FPUT model, compared to that of the Toda model. We extended this analysis to relate to a method for an irreversible energy dissipation process suggested by Onorato et al. [41] (four-wave and six-wave resonances in wave turbulence theory). Our preliminary results confirm the presence of resonances in the spectrum, but it is not clear these are those proposed by Oronato et al. [41]. Future work is anticipated on this point.

Turning our attention to the β-FPUT model, we explored the two different signs of β, something which is not interesting in the α-FPUT model because the α-FPUT Hamiltonian is symmetric under α→−α. The spectra for the β-FPUT model suggestively point to resonances leading to equipartition as well. We developed a procedure for calculating $t_{m}$ in the β-FPUT model, a very different task than for the α-FPUT model because the β-FPUT model is not the truncation of a non-trivial integrable model.

## Data Availability

Data available on request due to large file sizes. Contact authors for specific data questions.
